# Assessing the genomic feature of Chinese patients with ampullary adenocarcinoma: potential therapeutic targets

**DOI:** 10.1186/s12885-024-11949-9

**Published:** 2024-03-04

**Authors:** Zhang Dong, Wan Chong, Chen Chen, Li Qi, Li Mengke, Dou Minghui, Yuan Jiawei, Quan Longxi, Liu Hengchao, Jia Liu, Geng Zhimin

**Affiliations:** 1 Department of Hepatobiliary Surgery, the First Affiliated Hospital of Xi’an Jiaotong University, 277 West Yanta Road, Xi’an, Shaanxi Province 710061 China; 2 Precision Medicine Center, Yangtze Delta Region Institute of Tsinghua University, Jiaxing, Zhejiang China; 3 Department of Precision medicine, Accb Biotech.Ltd, Beijing, China

**Keywords:** Ampullary adenocarcinoma, Somatic, Germline, DDR, Actionable alteration

## Abstract

**Backgrounds:**

Ampullary adenocarcinoma (AMPAC) is a rare malignancy, treated as pancreatic or intestinal cancer based on its histologic subtype. Little is known about the genomic features of Chinese patients with AMPAC.

**Materials and methods:**

We enrolled 145 Chinese AMPAC patients in our local cohort and performed a compressive somatic and germline genetic testing using a 156 gene panel. Expression of PD-L1 (clone 28 − 8) was also assessed in tumor specimens from 64 patients.

**Results:**

The frequency of genetic alterations (GAs) in Chinese patients with AMPAC was found to be distinctive, with *TP53*, *KRAS*, *SMAD4*, *APC*, *CTNNB1*, *ARID1A*, and *CDKN2A* emerged as the most frequently mutated genes. Comparing with Western patients, significant differences were observed in the prevalence of *PIK3CA* and *ARID2*. Furthermore, the incidence of MSI-H was lower in the Chinese cohort, with only two patients identified as MSI-H. Conversely, 11 patients (8.27%) had pathogenic/likely pathogenic germline alterations, all of which were in the DNA damage response (DDR) pathway. In our cohort, 34.48% (22/64) of patients exhibited positive PD-L1 expression in tumor cells, and this expression was associated with GAs in *CTNNB1* and *BLM*. Importantly, over three-fourths of Chinese AMPAC patients in our study had at least one actionable GA, with more than one-fifth of them having actionable GAs classified as Level 3. These actionable GAs were primarily involved in the DDR and PI3K pathways. Notably, GAs in the DDR pathway were detected in both Chinese and Western patients, and regardless of their functional impact, these alterations demonstrated enhanced overall survival rates and higher tumor mutational burden (TMB) levels.

**Conclusion:**

These findings underscore the distinct genomic landscape of Chinese AMPAC patients and highlight the potential for targeted therapies based on the identified GAs.

**Supplementary Information:**

The online version contains supplementary material available at 10.1186/s12885-024-11949-9.

## Introduction

Ampullary adenocarcinoma (AMPAC) is a rare malignancy that originates within the ampulla of Vater. Its incidence is approximately 0.59 cases per 100,000, but has been steadily increasing over the past few decades [1]. Although AMPAC only accounts for 0.2% of gastrointestinal malignancies, it carries significant clinical importance due to its pathological variations and associated prognosis. The 5-year overall survival rate for AMPAC patients who undergo resection ranges from 35 to 50%, with outcomes heavily influenced by various clinical and histological factors, particularly tumor stage and treatment [2]. Despite nearly 80% of AMPAC cases being resectable at diagnosis, approximately half of these cases may experience disease recurrence [3]. For patients with advanced AMPAC, first-line systemic therapy typically involves chemotherapy, with the specific regimen selected based on the subtype [4]. However, the antitumor efficacy of chemotherapy, regardless of the treatment regimen, remains unsatisfactory [5]. Therefore, gaining a better understanding of the biological features of AMPAC may provide valuable insights for the development of more effective treatment options.

Biomarker-driven cancer therapy is at the forefront of precision medicine in oncology, and the advent of novel technologies, particularly next-generation sequencing (NGS), has exponentially expanded our knowledge of the genetic characteristics and actionable alterations in various cancer types [6]. However, due to its low incidence, current understanding of the genomic profile of AMPAC lags behind that of other gastrointestinal malignancies [7, 8]. Recent two major studies have shed important light on the genetic characteristics of AMPAC. Gingras et al. investigated the genomic features of 98 AMPAC cases and identified genomic similarities with bile duct cancer and duodenal adenocarcinomas [9]. AMPAC was characterized by a high prevalence of genomic alterations (GAs) in genes associated with the WNT pathway, as well as inactivating GAs in *ELF3*. Another study by Yachida et al. identified common GAs in *KRAS*, *TP53*, *CTNNB1*, *SMAD4*, *APC*, and *ELF3* in a cohort of 172 Japanese and American AMPAC patients [10]. However, it remains unknown whether there are genomic differences between Eastern and Western patients, and little is known about the genomic features of Chinese patients with AMPAC. The National Comprehensive Cancer Network (NCCN) guidelines recommend genetic testing for AMPAC patients, including genes such as *ALK*, *NRG1*, *NTRK*, *ROS1*, *FGFR2*, *RET*, *BRAF*, *BRCA1/2*, *KRAS*, and *PALB2* [2]. However, it is worth noting that this recommendation is directly adapted from that for pancreatic cancer, and the actual prevalence of these actionable alterations in AMPAC remains uncertain.

To better understanding of the genomic feature of Chinese AMPAC patients, we have enrolled 145 patients in our cohort to perform genomic profiling. We aimed to access (1) the germline and somatic genomic feature of Chinese AMPAC patients; (2) Genomic difference with the Western AMPAC patients; (3) actionable genomic alterations that may have targeted therapy choice.

## Materials and methods

### Patient enrollment

Subjects with AMPAC were enrolled in this study between April 2018 and January 2023. In our study, the diagnosis of AMPAC strictly adhered to the definition established by Adsay et al. [11]. and Reid et al [12]. Specifically, the tumors needed to have their epicenter located in the ampulla, including the lumen or walls of the distal ends of the common bile duct and/or pancreatic duct, or at the papilla of Vater. Additionally, no more than 75% of the tumor was allowed to be situated away from the ampulla area. All the samples included in this study were confirmed to be AMPAC, excluding those with tumors originating from the duodenum, pancreas, or common bile duct but involving the ampulla. To ensure accuracy and consistency, two investigators (CC and LMK) centrally examined all cases in the study. The additional inclusion criteria included that (1) patients were at least 18 years old; (2) have sufficient formalin-fixed, paraffin-embedded (FFPE) tumor tissue samples; (3) FFPE samples with tumor cell contents beyond 20%. This study was approved by the First Affiliated Hospital of Xi’an Jiaotong University and all enrolled patients had provided written informed consents by themselves or their legal guardians. Finally, a total of 145 patients were enrolled, and 133 of them were consented to conduct an accompanying germline testing.

### Immunohistochemistry

Immunohistochemistry analysis of 141 FFPE samples was successfully conducted to determine the histology type. This analysis primarily utilized markers such as MUC1 (clone ID: EP85), MUC2 (Ccp58), CDX2 (EP25), and CK20 (EP23, all bought from ZSGB-BIO., Beijing, China). The classification of histology followed the definition provided by Ang et al. [13]: (1) Intestinal-type was characterized by positive staining for CK20 or CDX2 or MUC2, and negative staining for MUC1, or positive staining for CK20, CDX2, and MUC2, regardless of the MUC1 result. (2) Pancreatobiliary-type was identified by positive staining for MUC1 and negative staining for CDX2 and MUC2. Cases that did not fit into either of these three categories were classified as “other.” Seventy-eight samples (53.79%) were classified as Intestinal-type, 39 (26.90%) as pancreatobiliary-type and sixteen tumors (11.03%) as mixed-type.

### NGS analysis

DNA was extracted from FFPE and peripheral blood mononuclear cells (PBMCs) using the FFPE DNA Automated Extraction Kit (Accbio, Jaxing, China) and TIANamp Blood DNA kit (DP348, TIANGEN BITOCH CO.,LTD, Beijing, China), respectively. The purified DNA was then quantified using the StepOnePlus System and Qubit 3.0 Fluorometer (Life Technologies, Inc.). At least 50 ng of DNA extracted from matched PBMCs and tumor samples was sheared with the Covaris E210 system (Covaris, Inc.) to obtain an average of 200 bp fragments. We prepared NGS libraries of tumor gDNA and matched germline gDNA using the Accel-NGS 2 S DNA Library Kit (Swift Biosciences, Inc.) and xGen Lockdown Probes kit (IDT, Inc.). The custom xGen Lockdown probe for targeting the exons and selecting intronic regions of 156 genes was synthesized by IDT, Inc (Supplementary Table 1). Paired-end sequencing (2 × 150 bp) was performed on a Novaseq 6000 platform (Illumina Inc) at CAP-authorized laboratory (Lifehealthecare, Hangzhou, China). The median coverage rate of the tumor DNA was 1800×. We then used the Burrows-Wheeler Aligner to align the raw sequencing data with the reference the hg19 genome, producing a binary alignment/map (BAM) file. After deduplication and local realignment, Genome Analysis Toolkit (GATK) and ANNOVAR software tools were utilized to identify single nucleotide variation (SNV)/ insertion and deletion (inDel) and annotate variants, respectively. Copy number variations were analyzed using CNVkit [14].

### Germline variant interpretation

Variants identified in germline DNA from PBMCs with allele fraction (AF) beyond 25% were determined as germline variants. Variants with frequency ≥ 1% in ExAC, 1000 Genomes or ESP6500 databases were removed. The interpretation of germline alterations followed the standards and guidelines of American College of Medical Genetics and Genomics and the Association for Molecular Pathology (ACMG/AMP) by two genetic counsellors independently [15].

### Somatic genomic alterations (GAs) interpretation

Variants identified in the tumor samples with AF beyond 1% and were not identified as germline variants were identified as tumor somatic GAs. The functional classification of each somatic GAs was followed the interpretation and reporting standards and guidelines recommended by the Association for Molecular Pathology, American Society of Clinical Oncology, and College of American Pathologists (ASCO/CAP) [16]. Furthermore, the biological function and therapeutic implications of each GA was annotated using OncoKB database (http://oncokb.org) which was granted recognition by the US Food and Drug Administration [17].

### Analysis of the tumor mutation burden (TMB) and microsatellite instability (MSI)

The tumor mutation burden for each sample was calculated based on a published and widely applied method [18]. As genetic testing was performed on the targeted panel with 158 genes in current study and TMB evaluation on whole exome sequencing (WES) is the gold standard, we analyzed the correlation between the TMB value measuring by the targeted panel and WES (xGen™ NGS, IDT, USA). The correlation value was 0.83 (Supplementary Fig. 1A), supporting the accuracy of the panel-based TMB value. MSI analysis was performed by mSINGS [19] using 24 informative microsatellite regions integrated in the same targeted panel. We analyzed the concordance in MSI results of 87 samples between this method and MSI Analysis System (Promega) with the widely acknowledged mononucleotide repeat markers (BAT-25, BAT-26, NR-21, NR-24, and MONO-27). The accuracy of the panel-based MSI evaluation was 0.97 (supplementary Fig. 1B).

### PD-L1 expression analysis

We performed immunohistochemistry to assess the expression of PD-L1 using the anti-PD-L1 monoclonal antibody (clone: 28 − 8, Dako). A pathologist scored the PD-L1 expression level of tumor cells based on the manufacturer’s instructions [20]. Samples with a tumor cell expression of over 1% were classified as PD-L1 positive.

### Comparison with the genomic profiles of western AMPAC patients

Genomic alterations data of western AMPAC patients (Ampullary Carcinoma (Baylor College of Medicine, Cell Reports 2016) [9]) from Memorial Sloan Kettering Cancer Center (MSKCC) database was downloaded from cBioPortal (http://www.cbioportal.org). In the meanwhile, GAs data from other two AMPAC datasets, including Wong et al. 2019 (*n* = 45) [21] and Harthimmer et al. 2019 (*n* = 54) [4] were collected in each paper.

### Statistical analysis

All analysis was performed using R software (version 4.1.2). All categorical variables were compared with the Fisher exact test. Overall survival was estimated with Kaplan–Meier methods and compared with the log–rank test. All tests were two-sided, and *P* < 0.05 was considered significant.

## Results

### Overview of the somatic alteration profile in Chinese AMPAC patients

The clinical and histopathologic parameters of enrolled patients were presented in Table 1. The median age at diagnosis of the enrolled patients was 64 years-old, and 93 of them (64.14%) were male. Their histologic subtypes were as followed: intestinal (*n* = 97 [66.90%]), pancreaticobiliary (*n* = 48 [33.10%]). All 145 AMPAC tumor samples were successfully conducted genetic testing.

**Table 1:** Clinical characteristics of Chinese patients with ampullary carcinoma

	Variable	No.	Ratio/Range
Age at diagnosis		Median	Range
		64	(35–82)
Gender			
	Female	52	35.86%
	Male	93	64.14%
Stage			
	IA	14	9.66%
	IB	15	10.34%
	IIA	13	8.97%
	IIB	55	37.93%
	IIIA	13	8.97%
	IIIB	15	10.34%
	IV	20	13.79%
Subtype			
	Intestinal	97	66.90%
	pancreaticobiliary	48	33.10%
Family cancer history			
	Yes	26	17.93%
	No	60	41.38%
	Unknown	59	40.69%

**Fig. 1:**
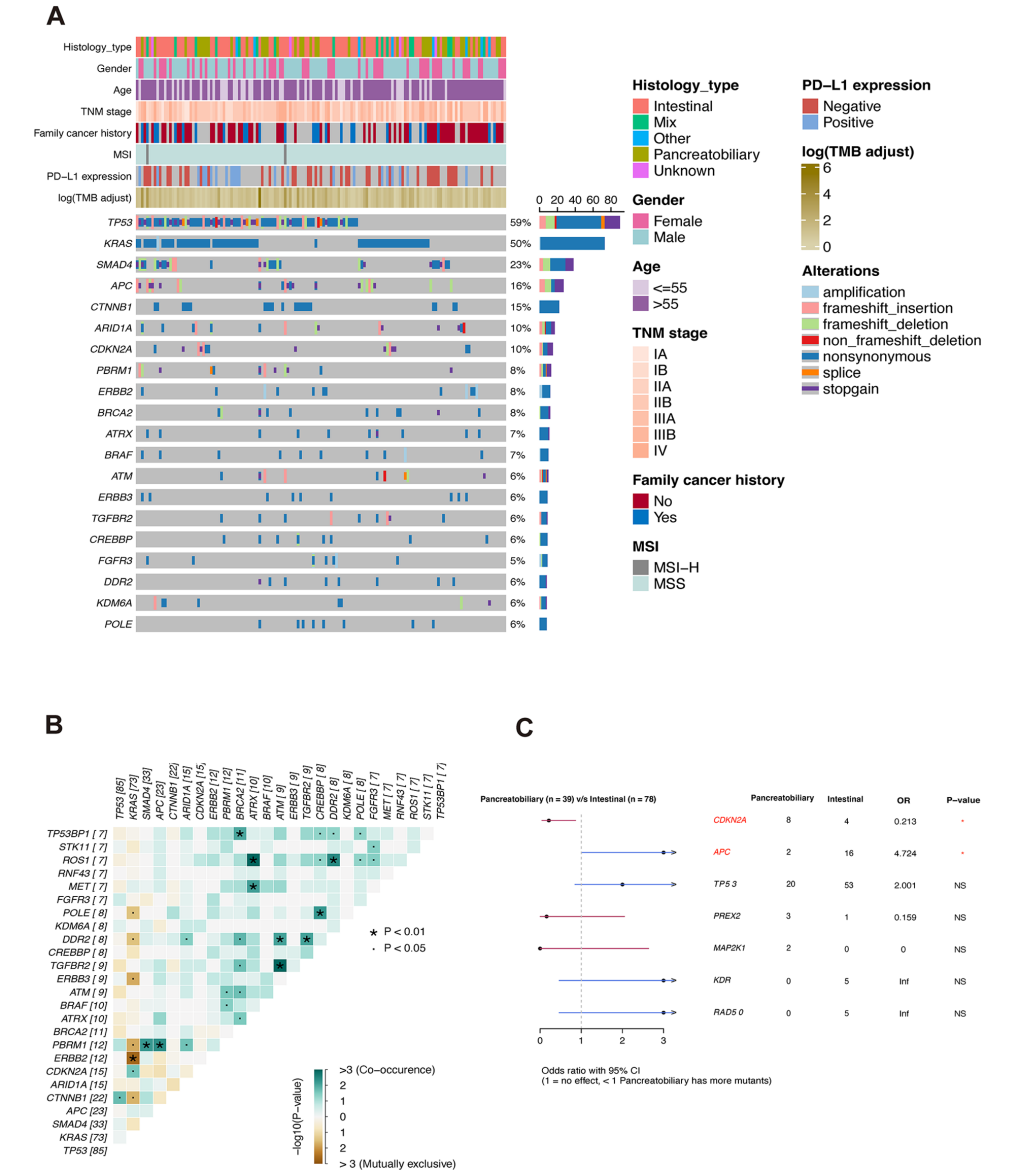
Overview of the somatic alteration profile in Chinese ampullary carcinoma patients. (**A**) Oncoprint figure: genomic alterations from 145 Chinese AMPAC patients are depicted. For each sample, mutation profile is shown in a column including 20 the most frequently altered genes. Genomic alterations included amplification, frameshift-insertion/deletion, non-frameshift-insertion/deletion, splice site, stopgain and nonsynonymous mutations. (**B**) The co-occurence or mutually exclusive relationship between altered genes in Chinese ampullary carcinoma patients. (**C**) The genomic disparities between intestinal-type and pancreatobiliary-type tumors in our cohort. *p* < 0.05; * *p* < 0.01

The frequency of GAs in Chinese patients with AC was found to be distinctive, with *TP53*, *KRAS*, *SMAD4*, *APC*, *CTNNB1*, *ARID1A*, and *CDKN2A* emerging as the most frequently mutated genes (Fig. 1A). Among these GAs, nonsynonymous alterations were the most common, with a high proportion of oncogenic alterations observed in *TP53*, *KRAS*, *CTNNB1*, and *ERBB2*. Interestingly, we observed a significant mutual exclusivity between *KRAS* and *ERBB2* alterations (Fig. 1B). Additionally, we identified significant co-occurrence of GAs in *BRCA2* and *TP53BP1*, *ROS1* and *ATRX*/*DDR2*, as well as *ATM* and *DDR2*/*ERBB3*. The median TMB value of Chinese AMPAC patients was determined to be 1.5 mutations/Mb. Among the patients, two with MSI-H showed TMB values of 28.0 and 8.0 mutations/Mb, respectively. Interestingly, one patient with a remarkably high TMB level of 248 mutations/Mb was found to be MSS, but multiple genetic alterations in DDR (DNA damage repair) were identified, particularly the *POLE*-P286R mutation, which may have induced the hypermutation phenotype. No significant difference in the frequency of GAs was observed between the intestinal and pancreaticobiliary subtypes, except for *CDKN2A* (prevalence intestinal vs. pancreaticobiliary: 4.12% vs. 18.75%, *p* < 0.05). Moreover, we investigated the genomic disparities between intestinal-type and pancreatobiliary-type tumors in our cohort (Fig. 1C). Our analysis revealed a significantly higher presence of *CDKN2A* alterations but a lower occurrence of *APC* alterations in the pancreatobiliary-type, which aligns with the findings reported by Shinichi Yachida et al. [10].

### Difference in the GAs between Chinese and western AMPAC patients

**Fig. 2:**
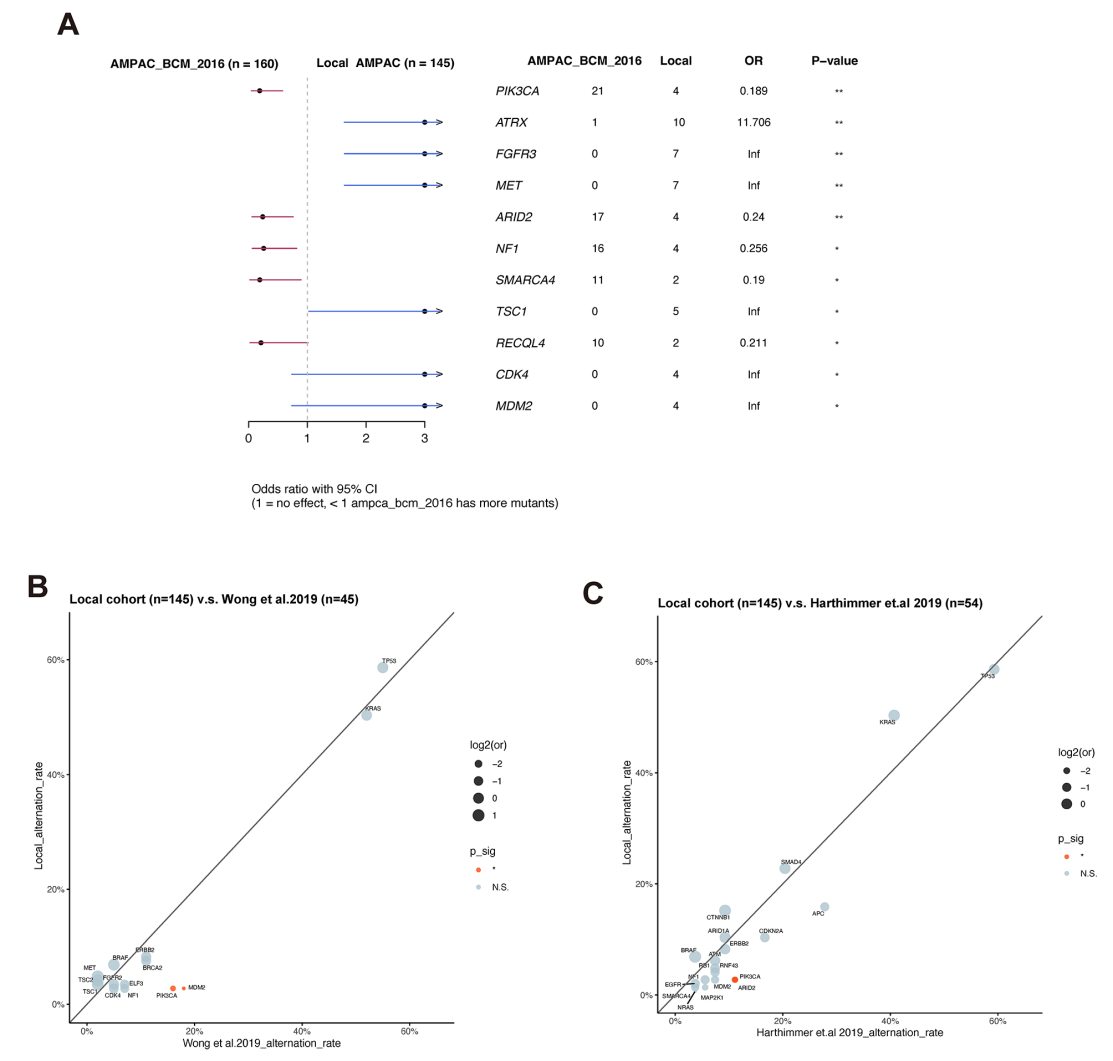
Difference in the genomic alterations between Chinese and western AMPAC patients. (**A**) Distribution of Genomic alterations with significant difference between AMPAC_BCM_2016 (*n* = 160) and local AMPAC cohort. Comparison of genomic alterations between local AMPAC cohort and Wong et al.2019 (**B**) and Harthimmer et al. 2019 (**C**) cohort. *p < 0.05; **p < 0.01; N.S.: non-significant

To gain a comprehensive understanding of the genomic characteristics of Chinese patients with AMPAC, we conducted a comparative analysis between their genomic profiles and those of Western AMPAC patients. This analysis revealed significant differences in the prevalence of eleven genes (Fig. 2A). Specifically, *PIK3CA*, *ARID2*, *NF1*, *SMARCA4*, and *RECQL4* exhibited higher alteration rates in the Western cohort, while *ATRX*, *FGFR3*, *MET*, *TSC1*, *CDK4*, and *MDM2* showed higher alteration rates in the Chinese cohort. It is important to note that the Chinese cohort had a higher proportion of stage IV patients and a lower number of stage III patients, which may potentially contribute to the observed differences (Supplementary Fig. 2 [9]). To further confirm the genomic differences between the Chinese and Western cohorts, we also compared the genomic findings from our local AMPAC cohort with those from other two AMPAC studies. Differences in *PIK3CA* and *ARID2* were also observed between the Chinese and Western AMPAC cohorts (Fig. 2B-C). These findings highlight the genomic disparities between Chinese and Western AMPAC patients.

### Germline alterations in Chinese AMPAC patients

In these unselected Chinese patients with AMPAC, 133 of them underwent germline genetic test. In total, 11 patients (8.27%) were identified with harboring pathogenic/likely pathogenic (P/LP) germline alterations (Table 2). Intriguingly, all these alterations were related to DNA damage repair, including 2 in mismatch repair and 9 in homologous recombination repair pathway. The leading germline mutated genes were *ATM*, *FANCA* and *PALB2* (each *n* = 2), followed by one patient each who carried mutations in *BRCA2*, *BARD1*, *MRE11A*, *MSH6*, and *MSH3*. The median age at diagnosis for those P/LP germline alterations carriers was 66-years old, which were not significantly differed to those with wildtype (64 years old). In 19 early onset patients (age at diagnosis below 55-years old), only 2 of them (10.52%) had P/LP alterations.

**Table 2:** Germline variants with clinical significance in Chinese patients with AMPAC

Patient NO.	Gender	Age	Stage	Gene	Exon	Coding seq change	Protein_change	HOM/ HET	Mutation_type	Clinical_significance	map_location	from	to
Patient014	Male	67	IV	BRCA2	11	c.6547delG	p.Glu2183fs	HET	frameshift-deletion	likely pathogenic	chr13:32915038	AG	A
Patient043	Male	66	IIB	PALB2	4	c.1317delG	p.Gly439fs	HET	frameshift-deletion	pathogenic	chr16:23646549	AC	A
Patient066	Female	47	IIIB	FANCA	4	c.367 C > T	p.Gln123Ter	HET	stopgain	likely pathogenic	chr16:89877396	G	A
Patient095	Female	74	IIIB	ATM	34	c.5170G > T	p.Glu1724Ter	HET	stopgain	likely pathogenic	chr11:108170605	G	T
Patient145	Female	66	IIB	ATM	57	c.8395_8404del TTTCA GTGCC	p.Phe2799fs	HET	frameshift-deletion	pathogenic	chr11:108214064	ATTTCA GTGCC	A
Patient123	Female	50	IB	MRE11	8	c.791 C > A	p.Ser264Ter	HET	stopgain	likely pathogenic	chr11:94204794	G	T
Patient009	Male	73	IIIA	MSH6	4	c.3037_3041del AAGAA	p.Lys1013fs	HET	frameshift-deletion	pathogenic	chr2:48028155	TGAAAA	T
Patient117	Male	76	IIIB	FANCA	32	c.3169 C > T	p.Gln1057Ter	HET	stopgain	likely pathogenic	chr16:89816208	G	A
Patient041	Male	68	IB	PALB2	4	c.246dupA	p.His83fs	HET	frameshift-insertion	likely pathogenic	chr16:23647620	G	GT
Patient132	Male	63	IIIB	MSH3	22	c.3083dupA	p.Tyr1028fs	HET	frameshift-insertion	likely pathogenic	chr5:80160713	T	TA
Patient030	Male	65	IIIB	BARD1	4	c.448 C > T	p.Arg150Ter	HET	stopgain	pathogenic	chr2:215646150	G	A

**HOM: Homogeneous; HET: Heterogeneous**

### PD-L1 expression and associated genomic feature

**Fig. 3:**
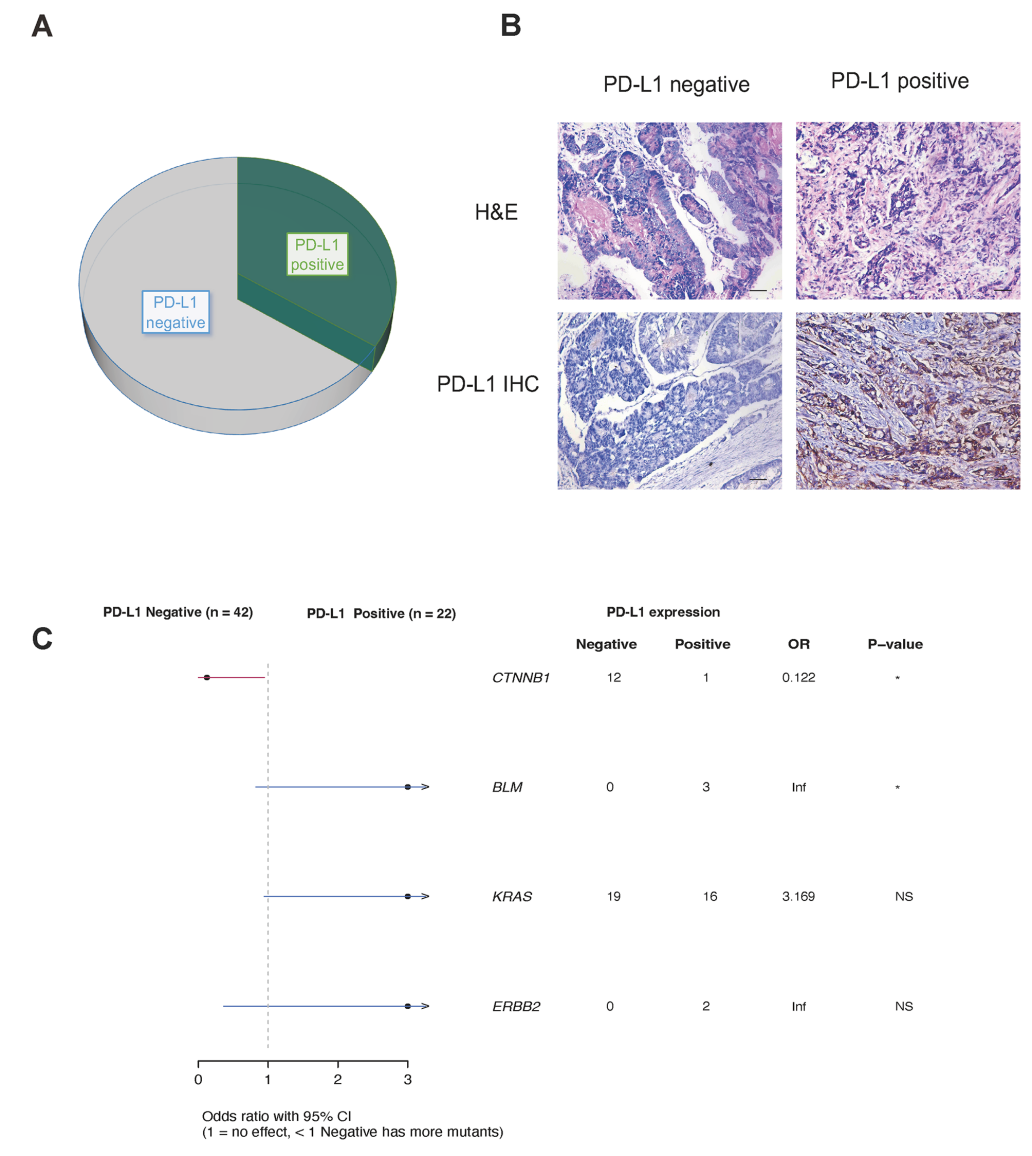
PD-L1expression and associated genomic feature. (**A**) Pie plot showing the ratio of PD-L1 positive samples in Chinese AMPAC cohort. (**B**) Representative images of PD-L1 positive and negative AMPAC samples. Scale bars: 200 μm. (**C**) Comparison of genomic difference between PD-L1 positive and negative AMPAC samples. * *p* < 0.05. NS: non-significant

Additionally, we also performed PD-L1 expression test in 64 AMPCA patients from our cohort, and 34.48% (22/64) of them had positive PD-L1 expression in tumor cells (TPS over 1%, Fig. 3A&B). In the meantime, we also assessed whether certain GAs are correlated with PD-L1 expression status and then analyzed the difference in the GAs between AMPAC patients with positive and negative PD-L1 expression. A significant difference in the prevalence of GAs in *CTNNB1* and *BLM* was identified with the association of PD-L1 expression (Fig. 3C).

### Actionable GAs

**Fig. 4:**
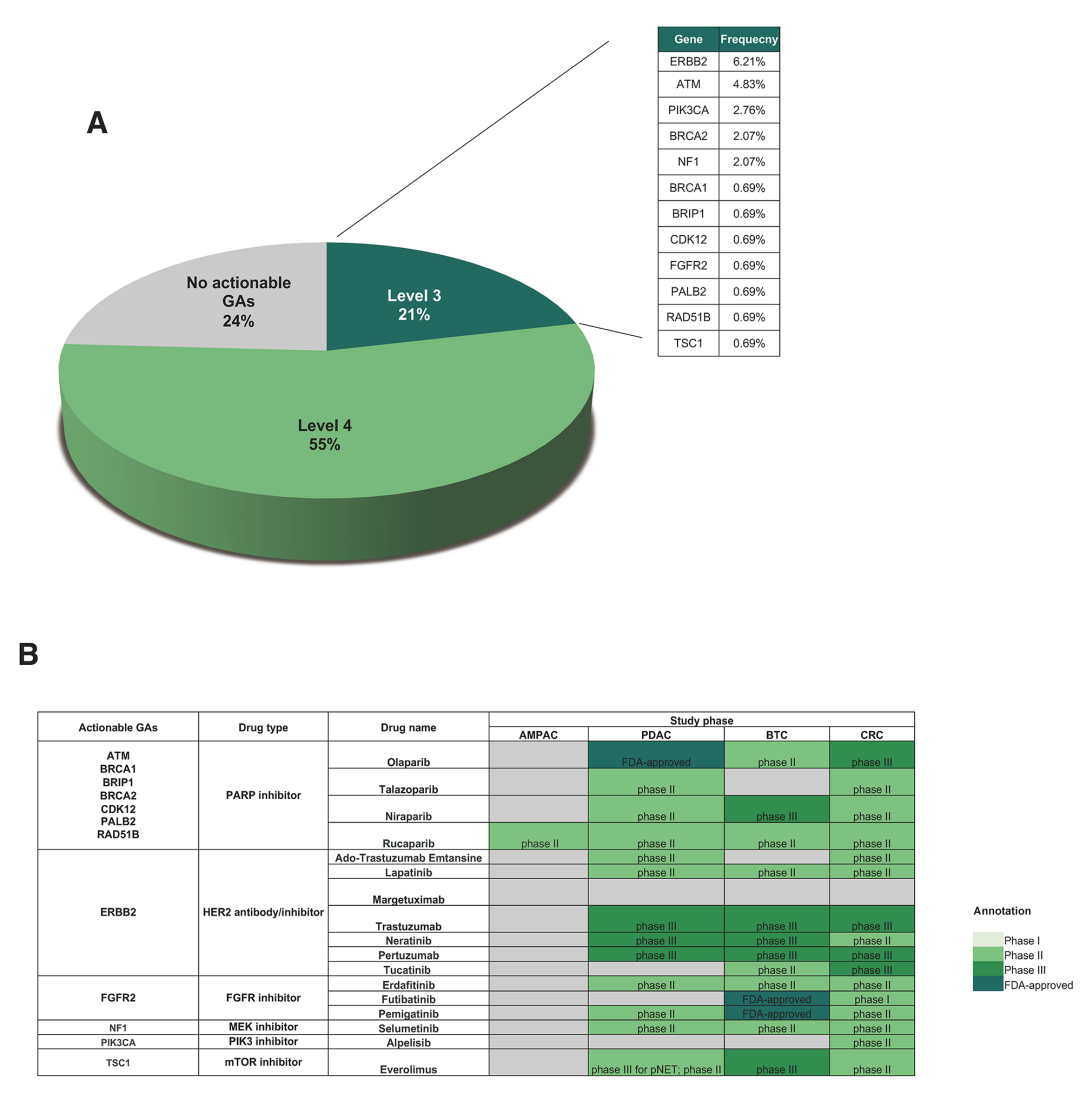
Identification of actionable genomic alterations (GAs) in Chinese patients with ampullary adenocarcinoma (AMPAC). (**A**) Prevalence of clinically actionable GAs and those classified as level 3. (**B**) Table presenting the approved and clinical trial data of matched therapies for level 3 GAs in AMPAC, pancreatic ductal adenocarcinoma (PDAC), biliary tract cancer (BTC), and colorectal cancer (CRC).

In our cohort, a total of 75.86% of Chinese patients with AMPAC were found to have at least one actionable GAs (Fig. 4A). Encouragingly, more than one-fifth of these patients had actionable GAs classified as Level 3, which refers to biomarkers with standard of care or investigational value in predicting response to FDA-approved or investigational drugs in other indications. The Level 3 actionable GAs were primarily enriched in the DNA damage response (DDR) pathway (*ATM*, *BRCA1*/2) and PI3K pathway (*PIK3CA*, *TSC1*), suggesting potential sensitivity to PARP inhibitors or PI3K inhibitors. Among the actionable GAs supported by Level 3 evidence, *FGFR2*, *BRCA1/2*, *PALB2*, and *ERBB2* have been recommended by the NCCN AMPAC guideline as markers for identifying potential candidates for matched anti-cancer therapy [22]. Additionally, all of these identified actionable GAs with Level 3 evidence align with the ESMO Scale for Clinical Actionability of molecular Targets (ESCAT) evidence tier III [23]. This alignment suggests that the matching of alterations with drugs is expected to have a positive impact on outcomes, as suggested by clinical trial data conducted in other tumor types or with similar molecular alterations. It is important to note that due to the rarity of AMPAC, none of these targeted drugs have been approved or extensively tested specifically for this indication (Fig. 4B). However, considering their approval or promising efficacy in pancreatic ductal adenocarcinoma (PDAC), biliary tract cancer (BTC), or bowel cancer, and the fact that AMPAC is managed similarly to these cancers, it is plausible that these targeted therapies may also be effective for patients with advanced AMPAC.

### DDR somatic GAs are prevalent in Chinese AMPAC patients and associated with prognosis

**Fig. 5:**
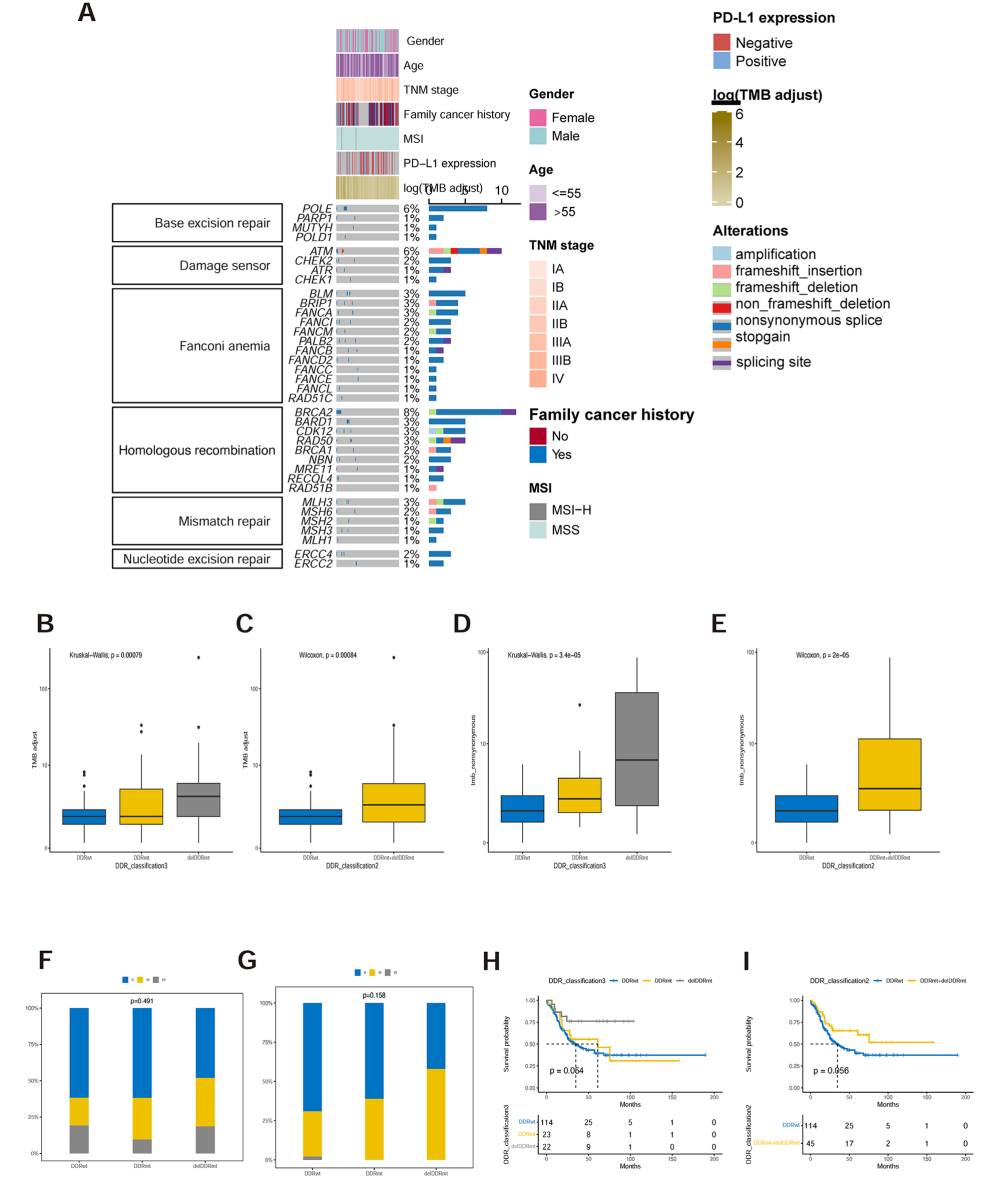
DNA damage repair (DDR) alterations are prevalent in Chinese AMPAC patients and associated with prognosis. (**A**) Distribution of DDR genomic alterations (DDR) in specific pathways. (**B**) Barplot showing the difference in the TMB value in DDRwt, DDRmt and delDDRmt groups in Chinese AMPAC cohort. (**C**) Barplot showing the difference in the TMB value between patients without and with any DDR alteration in Chinese AMPAC cohort. (**D**) Barplot showing the difference in the TMB value in DDRwt, DDRmt and delDDRmt groups in western AMPAC cohort. (**E**) Barplot showing the difference in the TMB value between patients without and with any DDR alteration in Western AMPAC cohort. Distribution of AMPAC patients with different clinical stages in Chinese (**F**) and Western AMPAC cohort (**G**). (**H-I**) overall survival by DDR alteration status. DDRmt: samples without identified DDR alteration; DDRmt: samples with DDR alterations with unknown function; delDDRmt: samples with deleterious DDR alterations

The DDR alteration was observed prominently among Chinese patients with AC, wherein 143 DDR genetic alterations were identified in 58 patients (40%). Among these, 41 deleterious DDR genetic alterations were detected in 30 patients (20.69%). Notably affected DDR genes included *BRCA2* (*n* = 12), *ATM* (*n* = 11), *POLE* (*n* = 8), as well as *BARD1* and *FANCA* (*n* = 6 each, as illustrated in Fig. 5A). The distribution of the identified DDR genetic alterations was also assessed based on pathways or mechanisms. Genes associated with Homologous Recombination, Fanconi Anemia, Damage Sensing, and Base Excision Repair were found to be most frequently implicated. Within our cohort, samples exhibiting deleterious DDR genetic alteration or any DDR genetic alteration displayed significantly higher levels of TMB (as depicted in Fig. 5B-C). Remarkably, this trend persisted in the western AC cohort, where patients with deleterious or any DDR genetic alterations also exhibited notably elevated TMB levels (as shown in Fig. 5D-E). Although there were no substantial discrepancies in disease stage between patients with and without DDR alterations in both the Chinese and western AC cohorts (Fig. 5F-G), it’s noteworthy that AMPAC patients harboring DDR alterations, irrespective of their functional impact, demonstrated enhanced overall survival rates compared to wildtype patients (as illustrated in Fig. 5H-I).

## Discussion

In the present study, we perform a comprehensive genomic study of 145 Chinese AMPAC patients from a single center.

The NCCN guideline recommend patients diagnosed with AMPAC or with a positive cancer history to take genetic testing for detecting genes involved for hereditary cancer syndromes [2]. The recommend genes, for instance, *ATM*, *BRCA1*, *BRCA2*, *CDKN2A*, *MLH1*, *MSH2*, *MSH6*, *PALB2*, *PMS2*, *STK11*, and *TP53*, are mainly involved with the hereditary pancreatic cancers. In current study, we found 8.27% of the Chinese AMPAC patients harbored P/LP germline variants, which were all in the DDR pathways. In a small cohort of mainly Caucasian AMPAC patients, the incidence of P/LP germline variants was 18% (8/44), and these variants were in *BRCA2*, *ATM*, *RAD50* and *MUTYH* [21]. However, this high ratio may be biased because the limited sample size. Intriguingly, we identified a AMPCA patient harbored *MSH3* P/LP germline with notable cancer family history, that his father was diagnosed with bladder cancer and his brother had rectal cancer. Unlike the canonical genes involved in the DNA mismatch pathway (*MLH1*, *MSH2*, *MSH6*, and *PMS2*), there is no direct evidence suggesting an association between MSH3 and Lynch syndrome. Previous studies have shown that loss of *MSH3* leads to an enrichment of ≥ 2 bp indel alterations and longer indels in malignant cells, which increases the risk of colorectal cancer [24, 25]. This may be due to the dysfunction of the MSH2-MSH3 (MutSβ) complex in cells, which is primarily responsible for repairing indel loops instead of base-base mismatches [26].

We identified several genomic differences between Chinese and Western AMPAC patients, particularly the consistent differences observed in *PIK3CA* and *ARID2*. However, to date, there have been no studies investigating the biological function of *PIK3CA* and *ARID2* specifically in AMPAC. *ARID2*, which encodes a subunit of the PBAF complex involved in chromatin remodeling, has been detected as a novel tumor suppressor in other cancers such as PDAC, CRC, and BTC [27–29]. Alterations in *ARID2* in bowel cancer have been associated with worse clinical outcomes and promoted proliferation and metastasis of malignant cells [28]. Additionally, *ARID2* is implicated in tumor immunology, particularly T cell cytotoxicity. Loss of *ARID2*, along with other genes in the SWI/SNF chromatin remodeling complex, may sensitize tumor cells to immunotherapy [30]. The PI3K-AKT signaling pathway has been shown to limit T cell recognition and the killing of cancer cells in PDAC, while downregulation of PIK3CA has been found to enhance T cell infiltration and promote tumor regression [31]. The difference in the prevalence of *ARID2* alterations in Chinese AMPAC patients may have implications for prognosis and therapeutic efficacy. Further research is needed to elucidate the specific roles of *PIK3CA* and *ARID2* in AMPAC and to explore how their alterations may influence disease progression and response to treatment in this particular patient population.

Previous evidence on the benefit of immunotherapy in treating AMPAC is poor. Cardin et al. has observed clinical benefit in a small cohort of AMPAC patients, but has to close the study because of the slow enrollment [32]. The MSI, TMB and PD-L1 level are the widely-acknowledged biomarkers associated with the response of immunotherapy [33]. In western AMPAC patients, the incidence of MMR-deficiency (MMRd) was higher than those with colorectal cancer. Previous studies found that 18% (23/127) of western patients with AMPAC had MMRd as evaluated by IHC [34]. However, in current study, we only identified two AMPAC patients (1.38%) with MSI-H, which was significantly lower than the incidence in the western patients (8.75%, *p* < 0.01) [35]. In a meanwhile, in a European cohort compromised of 59 AMPAC cases, only 8.2% (4/49) of them were MSI-H, which all had a loss of MLH1 an or PMS2 protein in immunohistochemical analysis [4]. Wong et al. found only 4.55% (2/44) of AMPAC patients were considered as MSI-H which were caused by somatic GAs in MMR genes [21]. A previous study also found a low incidence of MSI-H in Chinese with duodenum cancer was 3.2% (8/243) [36]. The PD-L1 expression level of patients with AMPAC has not been comprehensively evaluated, especially for Chinese patients. Previous study found 26.9% of invasive APC and 6.0% of ampullary dysplastic samples were PD-L1 positive, and PD-L1 expression samples were mainly intestinal-type and poorly differentiated [37]. Most of the PD-L1-positive tumors (seven of 10) were intestinal-type and poorly differentiated (G3). PD-L1 expression was associated with the MMR status. In 22 patients with MMRd, only 18.18% of them had positive PD-L1 expression in tumor cells (TPS$\ge$1%); however, 10 of them had combined positive score (CPS) over 1%, which indicated a correlation between MMRd and PD-L1 expression in immune cells [34]. In concordance with our result, previous study has found dysfunction of WNT pathway, especially frequently-altered *CTNNB1* in patients with AMPAC [9]. Even though multiple targeted drugs have been developed, there still lacks effective personalized medicine targeted at *CTNNB1* and/or WNT pathway currently. However, multiple studies have suggested the *CTNNB1* GAs as a negative predictive biomarker for immunotherapy, which shaped an immunosuppressive tumor microenvironment with depleting of T cells [38]. In our study, we identified for the first time, a negative correlation between *CTNNB1* GAs and PD-L1 expression in AMPAC, which was in consistent with previously-noted feature in other cancer types [39].

Conducting of an umbrella study to prove the reasonability of biomarker-matched therapy in AMPAC is problematic due to the rarity of the disease and even lower incidence of actionable GAs in it. However, the real world study, such as “Know your tumor” in pancreatic cancer has proved the survival benefits in biomarker-matched therapy [40]. In this study, we found over 75% of Chinese AMPAC patients with actionable alterations and over 20% of them had actionable GAs in Level 3, which were standard of care or investigational biomarkers predictive of response to an FDA-approved or investigational drug in another indications. Another study has found a similar frequency (70%) of actionable GAs in 97 Indian AMPAC patients [41]. Adoption of anti-HER2 therapy, ado-trastuzumab emtansine, has achieved partial response for approximately 6 months for a AMPAC patient with *ERBB2* amplification [21]. For patients with DDR GAs which may confer sensitivity to PARP inhibitors, a phase II, umbrella clinical trial that evaluate the efficacy of the combination of ATR inhibitor (AZD6738) with PARP inhibitor or immunotherapy for advanced biliary tract cancer, including AMPAC, is ongoing [42].

There are several limitations to our study that should be acknowledged. Firstly, we utilized a targeted gene panel for genetic testing instead of WES, which resulted in some missing genomic information due to the limited number of genes included. Secondly, we did not test for *NTRK1*, *NTRK2*, and *NTRK3* fusions, which are recommended pan-cancer druggable biomarkers according to the NCCN guidelines. The exclusion of *NTRK* genes was due to the limitations of DNA-based sequencing methods in detecting such fusions [43]. Additionally, despite our efforts to increase the sample size in our cohort, the study still had a limited number of samples due to the rarity of AMPAC. Moreover, although we identified actionable genetic alterations, these patients did not receive matched therapy based on these biomarkers prior to the finish of the current study. Therefore, it remains unknown whether these identified biomarkers can truly serve as predictive biomarkers for guiding matched targeted therapy in AMPAC. Lastly, we did not collect comprehensive follow-up data to correlate genomic findings with the prognosis of AMPAC. Hence, further studies with larger sample sizes are necessary to investigate the genomic characteristics of Chinese patients with AMPAC, particularly those that may differ from Western patients.

In summary, we conducted a genomic analysis on the largest cohort of Chinese AMPAC patients to date and have made the data available for future drug development and applications.

### Electronic supplementary material

Below is the link to the electronic supplementary material.

Supplementary Material 1

## Data Availability

The datasets generated and/or analyzed during the current study are available in the National Genomics Data Center, China National Center for Bioinformation/Beijing Institute of Genomics, Chinese Academy of Sciences under the access number is HRA004743 (https://ngdc.cncb.ac.cn/search/?dbId=hra&q=HRA004743).
