# Supplementary material for: Assessing the genomic feature of Chinese patients with ampullary adenocarcinoma: potential therapeutic targets
Source: BMC Cancer. 2024 Mar 4;24:286. doi: 10.1186/s12885-024-11949-9 (PMC10910796; doi:10.1186/s12885-024-11949-9)

Supplementary information

Supplementary table 1: Genes tested in the study

Supplementary figure 1. (A) Correlation analysis between the TMB results between the targeted panel and WES. (B) ROC curve of the MSI result determined by 24 informative microsatellite regions integrated in the targeted panel. TMB: tumor mutation burden; WES: whole exome sequencing; ROC: receiver operating characteristic; MSI: microsatellite instability

Supplementary figure 2. Comparison of the clinical data between Chinese and western AMPAC_BCM_2016 cohort.

Supplementary table 1: Genes tested in the study

Gene	Related pathway	Gene function
ABL1	RAS-RAF-RTK	OG
ALK	RAS-RAF-RTK	OG
APC	WNT	TSG
ARAF	RAS-RAF-RTK	OG
ARID1A	Chromatin-remodeling	TSG
ARID1B	Chromatin-remodeling	TSG
ARID2	Chromatin-remodeling	TSG
ATM	DNA damage repair	TSG
ATR	DNA damage repair	TSG
ATRX	DNA damage repair	TSG
AXIN1	WNT	TSG
AXIN2	WNT	TSG
B2M	cell cycle	TSG
BAP1	Chromatin-remodeling	TSG
BARD1	DNA damage repair	TSG
BLM	DNA damage repair	TSG
BMPR1A	TGF-Beta	TSG
BRAF	RAS-RAF-RTK	OG
BRCA1	DNA damage repair	TSG
BRCA2	DNA damage repair	TSG
BRIP1	DNA damage repair	TSG
CASP8	Cell cycle	TSG
CCND1	Cell cycle	OG
CCNE1	Cell cycle	OG
CDH1	EMT	TSG
CDK12	DNA damage repair	TSG
CDK4	Cell cycle	OG
CDKN1B	Cell cycle	TSG
CDKN2A	Cell cycle	TSG
CHD1	Chromatin-remodeling	TSG
CHEK1	DNA damage repair	TSG
CHEK2	DNA damage repair	TSG
CREBBP	NOTCH	TSG

CTNNB1	WNT	OG
DDR2	DNA damage repair	TSG
DNMT3A	chromatin-remodeling	TSG
EGFR	RAS-RAF-RTK	OG
ELF3	transcriptional activator	TSG
EPHA3	ephrin	TSG
EPHA5	ephrin	TSG
EPHB1	ephrin	TSG
ERBB2	RAS-RAF-RTK	OG
ERBB3	RAS-RAF-RTK	OG
ERBB4	RAS-RAF-RTK	OG
ERCC2	DNA damage repair	TSG
ERCC4	DNA damage repair	TSG
ERRFI1	RAS-RAF-RTK	TSG
ESR1	transcription factor	OG
EZH2	Chromatin-remodeling	OG
FANCA	DNA damage repair	TSG
FANCB	DNA damage repair	TSG
FANCC	DNA damage repair	TSG
FANCD2	DNA damage repair	TSG
FANCE	DNA damage repair	TSG
FANCI	DNA damage repair	TSG
FANCL	DNA damage repair	TSG
FANCM	DNA damage repair	TSG
FBXW7	NOTCH	TSG
FGF19	RAS-RAF-RTK	OG
FGF3	RAS-RAF-RTK	OG
FGF4	RAS-RAF-RTK	OG
FGFR1	RAS-RAF-RTK	OG
FGFR2	RAS-RAF-RTK	OG
FGFR3	RAS-RAF-RTK	OG
FGFR4	RAS-RAF-RTK	OG
FH	tricarboxylic acid cycle	TSG
FLCN	PI3K	tsg
FLT1	Angiogenesis	OG

FLT3	Angiogenesis	OG
FLT4	Angiogenesis	OG
GEN1	DNA damage repair	TSG
GNA11	RAS-RAF-RTK	OG
GNAQ	RAS-RAF-RTK	OG
GNAS	RAS-RAF-RTK	OG
HDAC4	Chromatin-remodeling	TSG
IDH1	tricarboxylic acid cycle	OG
IDH2	tricarboxylic acid cycle	OG
JAK1	RAS-RAF-RTK	OG
JAK2	RAS-RAF-RTK	OG
JAK3	RAS-RAF-RTK	OG
KDM6A	Chromatin-remodeling	TSG
KDR	Angiogenesis	OG
KEAP1	NRF3	TSG
KIT	Angiogenesis	OG
KRAS	RAS-RAF-RTK	OG
MAGI2	TGF-Beta	TSG
MAP2K1	RAS-RAF-RTK	OG
MAP2K2	RAS-RAF-RTK	OG
MAP2K4	RAS-RAF-RTK	OG
MDM2	TP53	OG
MDM4	TP53	OG
MET	RAS-RAF-RTK	OG
MITF	WNT/RAS-RAF-RTK	OG
MLH1	DNA damage repair	TSG
MLH3	DNA damage repair	TSG
MRE11	DNA damage repair	TSG
MSH2	DNA damage repair	TSG
MSH3	DNA damage repair	TSG
MSH6	DNA damage repair	TSG
MTOR	PI3K	OG
MUTYH	DNA damage repair	TSG
NBN	DNA damage repair	TSG
NF1	RAS-RAF-RTK	OG

NOTCH2	NOTCH	TSG
NRAS	RAS-RAF-RTK	OG
PALB2	DNA damage repair	TSG
PARP1	DNA damage repair	TSG
PBRM1	Chromatin-remodeling	TSG
PDGFRA	RAS-RAF-RTK	OG
PDGFRB	RAS-RAF-RTK	OG
PIK3CA	PI3K	OG
PIK3CG	PI3K	OG
PIK3R1	PI3K	TSG
PMS2	DNA damage repair	TSG
POLD1	DNA damage repair	TSG
POLE	DNA damage repair	TSG
PPM1D	DNA damage repair	OG/TSG
PPP2R2A	DNA damage repair	TSG
PREX2	RAC	Unknown
PRKD1	PKD	Unknown
PTCH1	Hedgehog	TSG
PTEN	PI3K	TSG
PTPN11	RAS-RAF-RTK	OG
PTPRD	RAS-RAF-RTK	TSG
PTPRT	RAS-RAF-RTK	TSG
RAD50	DNA damage repair	TSG
RAD51B	DNA damage repair	TSG
RAD51C	DNA damage repair	TSG
RB1	Cell cycle	TSG
RBM10	unknkown	OG/TSG
RECQL	DNA damage repair	TSG
RECQL4	DNA damage repair	TSG
RET	RAS-RAF-RTK	OG
RIT1	RAS-RAF-RTK	OG
RNF43	WNT	TSG
ROS1	RAS-RAF-RTK	OG
SDHB	HIF	TSG
SDHC	HIF	TSG

SETD2	Chromatin-remodeling	TSG
SMAD4	TGF-Beta	TSG
SMARCA4	Chromatin-remodeling	TSG
SMARCB1	Chromatin-remodeling	TSG
SMO	Hedgehog	TSG
SOX9	Multiple	OG/TSG
SPTA1	DNA replication	Unknown
STAG2	DNA replication	TSG
STK11	PI3K	TSG
TERT	Multiple	OG
TGFBR1	TGF-Beta	TSG
TGFBR2	TGF-Beta	TSG
TOP1	DNA damage repair	OG
TP53	TP53	TSG
TP53BP1	TP53	TSG
TSC1	PI3K	TSG
TSC2	PI3K	TSG
WRN	DNA damage repair	TSG

TSG: tumor suppressor gene; OG: oncogene

Supplementary figure 1. (A) Correlation analysis between the TMB results between the targeted panel and WES. (B) ROC curve of the MSI result determined by 24 informative microsatellite regions integrated in the targeted panel. TMB: tumor mutation burden; WES: whole exome sequencing; ROC: receiver operating characteristic; MSI: microsatellite instability

A

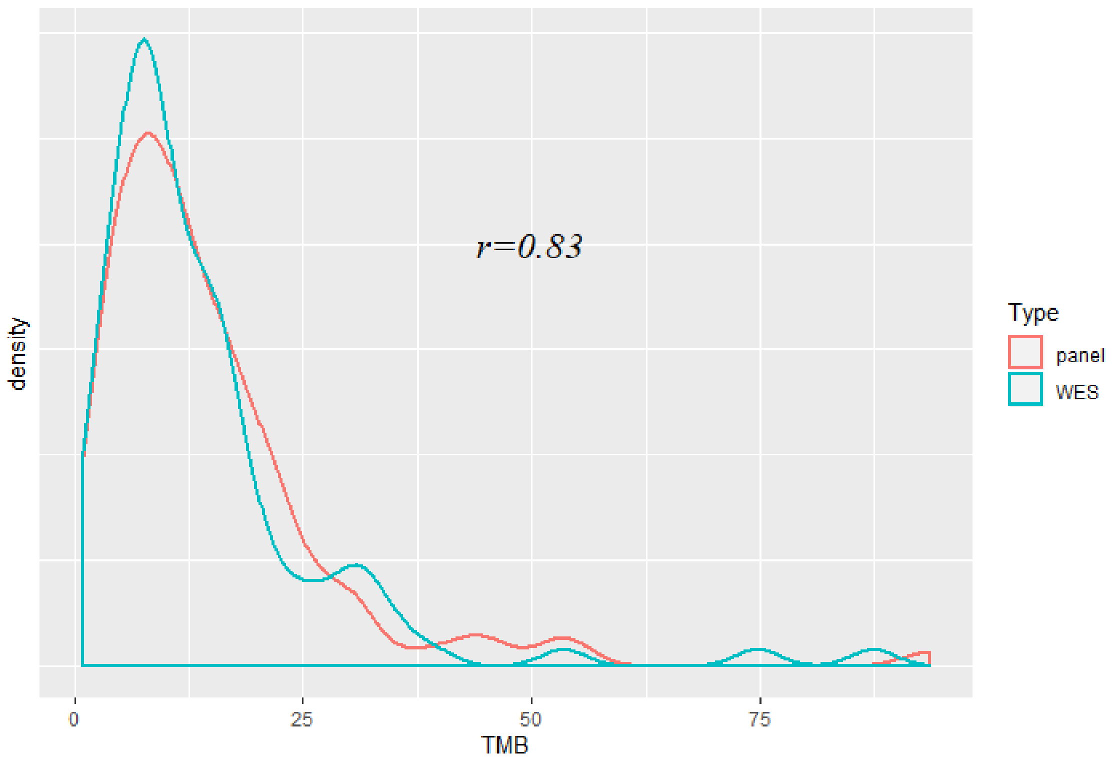

B

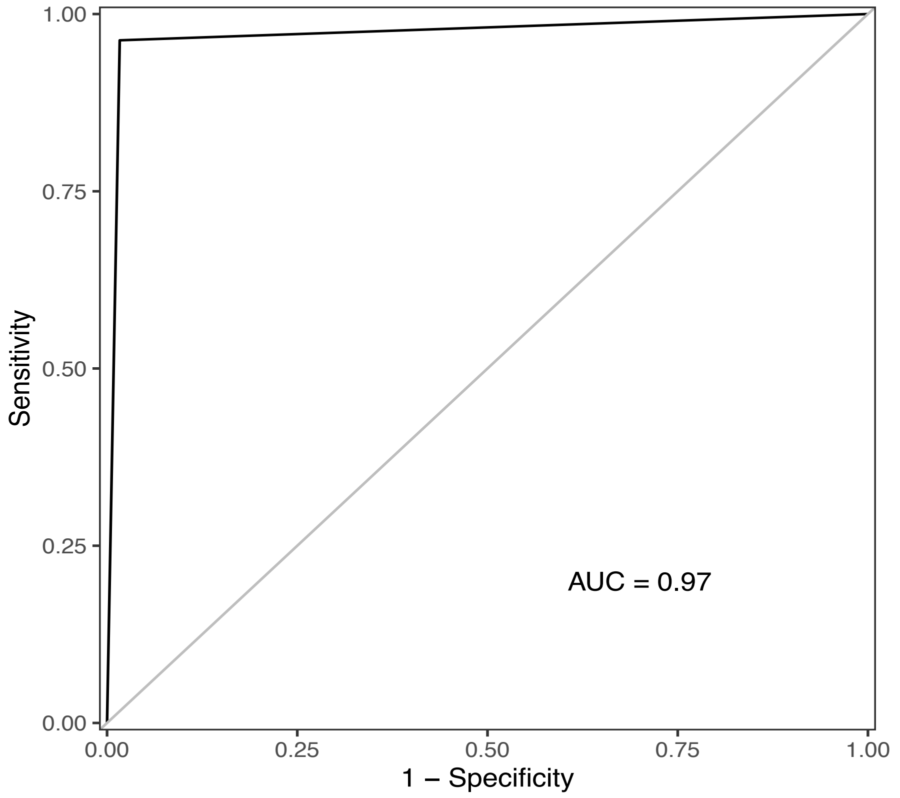

Supplementary figure 2. Comparison of the clinical data between Chinese and western AMPAC_BCM_2016 cohort.

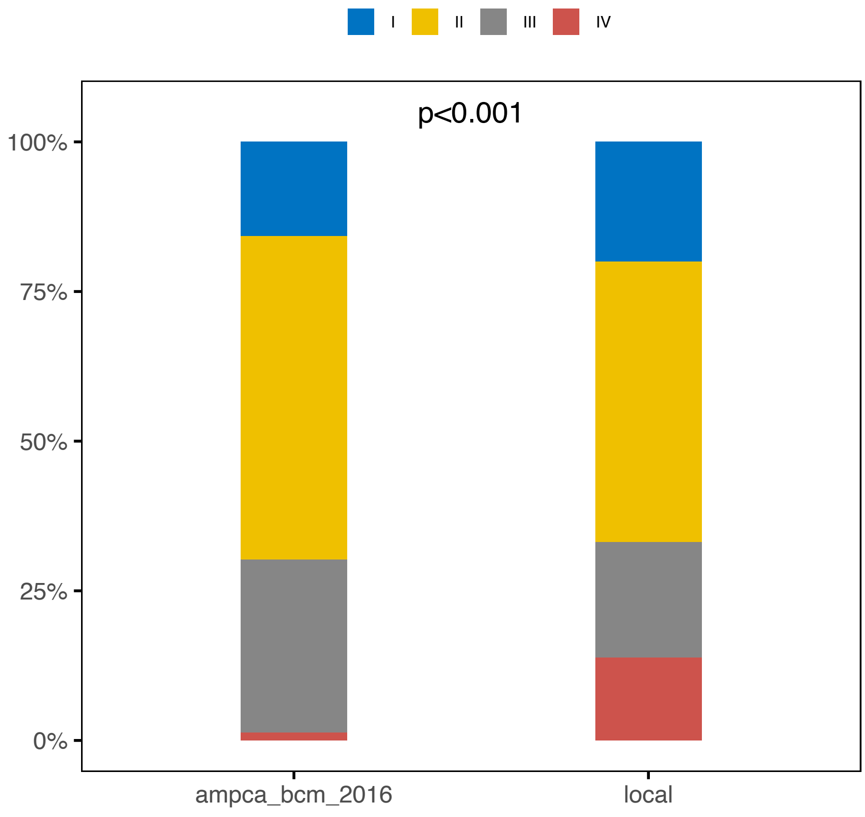

Supplement: Supplementary file 1 — Supplementary Material 1 [file 12885_2024_11949_MOESM1_ESM.pdf]
